# Identification and Comparison of Receptor Binding Characteristics of the Spike Protein of Two Porcine Epidemic Diarrhea Virus Strains

**DOI:** 10.3390/v8030055

**Published:** 2016-02-23

**Authors:** Feng Deng, Gang Ye, Qianqian Liu, Muhammad Tariq Navid, Xiaoli Zhong, Youwen Li, Chunyun Wan, Shaobo Xiao, Qigai He, Zhen F. Fu, Guiqing Peng

**Affiliations:** 1 State Key Laboratory of Agricultural Microbiology, Huazhong Agricultural University, Wuhan 430070, China; dengfeng207@163.com (F.D.); ehuangliu@webmail.hzau.edu.cn (G.Y.); liuqq563705@163.com (Q.L.); dr_tariqnaveed@hotmail.com (M.T.N.); Shirleyandyueyue@163.com (X.Z.); lyw_lk@163.com (Y.L.); vet@mail.hzau.edu.cn (S.X.); 2 College of Veterinary Medicine, Huazhong Agricultural University, Wuhan 430070, China; supwzfu@163.com; 3 Departments of Pathology, College of Veterinary Medicine, University of Georgia, Athens, GA 30602, USA; 4 The Cooperative Innovation Center for Sustainable Pig Production, Huazhong Agricultural University, Wuhan 430070, China

**Keywords:** PEDV, S protein, RBD, pAPN, CV777 strain, CHGD-01 strain, sugar

## Abstract

Porcine epidemic diarrhea virus (PEDV), a member of *Alphacoronavirus*, has caused huge economic losses for the global pork industry recently. The spike (S) protein mediates PEDV entry into host cells. Herein, we investigated the interactions between the S protein and its receptor porcine aminopeptidase N (pAPN) or co-receptor sugars. The C-terminal domain (CTD) of the S1 domain is bound to pAPN. The prototype strain demonstrated similar receptor-binding activity compared with the variant field isolate. Three loops at the tips of the β-barrel domains did not play crucial roles in the PEDV S-pAPN association, indicating that PEDV conforms to a different receptor recognition model compared with transmissible gastroenteritis virus (TGEV), porcine respiratory CoV (PRCV), and human coronavirus NL63 (HCoV-NL63). The N-terminal domain (NTD) of the PEDV S1 domain could bind sugar, a possible co-receptor for PEDV. The prototype strain exhibited weaker sugar-binding activity compared with the variant field isolate. Strategies targeting the receptor binding domain (RBD) may be helpful for developing vaccines or antiviral drugs for PEDV. Understanding the differences in receptor binding between the prototype and the variant strains may provide insight into PEDV pathogenesis.

## 1. Introduction

Coronaviruses are enveloped, single-stranded, positive-sense RNA viruses. They are globally important infectious agents that are associated with respiratory, digestive, and neurological diseases in humans and animals [1]. The *Coronaviridae* family consists of four genera known as the *α*-, *β*-, *γ*-, and *Deltacoronaviruses* [1,2]. These viruses use a variety of cellular receptors and co-receptors, including proteins and sugars. Severe acute respiratory syndrome coronavirus (SARS-CoV) in the *Betacoronavirus* genus uses angiotensin-converting enzyme 2 (ACE2) as its receptor, and its receptor-binding domain (RBD) is located within CTD of the S1 domain [3]. ACE2 is also a receptor for human coronavirus NL63 (HCoV-NL63), in which three discontinuous fragments located within CTD of the S1 domain are responsible for receptor binding [4]. No structural homology has been found between the SARS and HCoV-NL63 RBDs, but they recognize the same receptor (ACE2). Meanwhile, their RBDs share the same receptor recognition hotspots [5]. The RBD of transmissible gastroenteritis virus (TGEV) is located within CTD of the S1 domain. The RBD crystal structures of TGEV and HCoV-NL63 show similarities, but they use different receptors [2]. The RBDs of murine hepatitis virus (MHV) and bovine coronavirus (BCoV) in the same genus of *Betacoronaviruses* are located in the N-terminus of the S1 domain. However, carcinoembryonic antigen cell adhesion molecule (CEACAM) and sugar serve as the receptors for MHV and BCoV, respectively, despite their high sequence homology [6,7].

PEDV belongs to the *Alphacoronavirus* genus and can cause an acute and highly contagious enteric disease. Its clinical symptoms include watery diarrhea, severe enteritis, vomiting, and weight loss; the mortality rate can reach 50%–90% in suckling piglets [8,9,10]. In 1977, Pensaert isolated a new coronavirus-like particle associated with diarrhea from Belgian swine breeding farms. This virus was different from TGEV and was designated CV777 [11]. Subsequently, the disease was reported in the United Kingdom, Germany, Canada, France, Switzerland, Hungary, and Italy. Since 2010, massive PED outbreaks have been reported in Asia. In April 2013, there was a sudden occurrence of PED disease that rapidly spread across the United States, causing high rates of death among piglets [8]. Currently, PED is considered a pandemic disease that causes substantial economic losses to pork producers all over the world.

**Figure 1:**
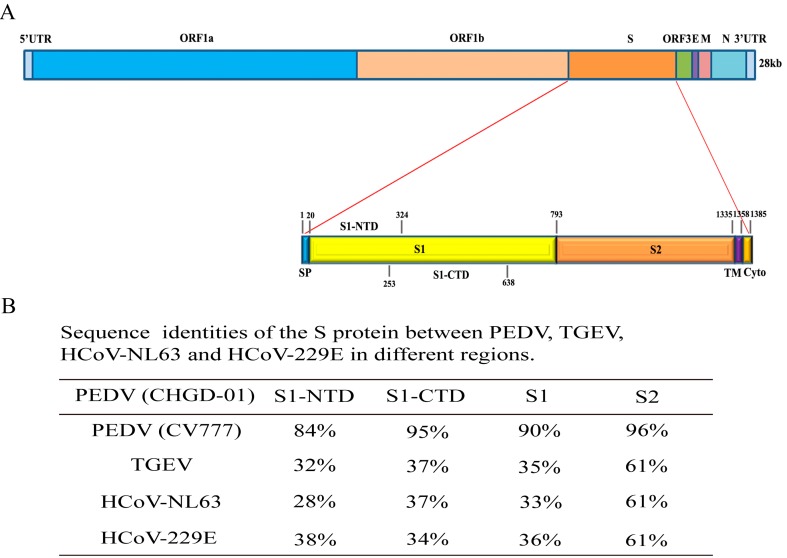
The S protein of PEDV. (**A**) Schematic diagram of the PEDV genome organization and the deduced domains of the S protein, including the signal peptide (SP); S1 region, which includes S1-NTD (residues 21–324) and S1-CTD (residues 253–638); S2 region; transmembrane domain (TM); and cytoplasmic tail (Cyto); (**B**) Sequence identities of the S protein between PEDV, TGEV, HCoV-NL63, and HCoV-229E in different regions. The sequences of the S1-NTD, S1-CTD, S1 and S2 regions of the PEDV CHGD-01 strain were compared with those of the corresponding regions of the PEDV CV777 strain, TGEV, HCoV-NL63, and HCoV-229E.

The genome of PEDV contains 28 kb which encodes the polyproteins ORF1a and ORF1b, S, open reading frame 3 (ORF3), envelope (E), membrane (M), and nucleocapsid (N) proteins [12,13]. The PEDV S protein is a homotrimeric membrane glycoprotein that contains a signal peptide (residues 1–20); an S1 region (residues 21–793) that mediates the attachment of virus particles to the cell surface receptor; an S2 region (residues 794–1385) that mediates virus fusion to host cells; a transmembrane domain (residues 1335–1358) and a cytoplasmic tail (residues 1359–1385); the S1 region contains two subdomains, an NTD (residues 21–324) and a CTD (residues 253–638) [14,15] (Figure 1A). Previous studies have confirmed that porcine aminopeptidase N (pAPN) acts as a receptor for PEDV entry into target cells [16,17,18]. All characterized coronavirus S1 domain contain an RBD that is responsible for interactions with the cellular receptors for viral attachment. Lee determined that the N-terminus of the PEDV S1 domain was necessary for receptor binding using a co-immunoprecipitation assay; the minimal binding region was confirmed to be located within residues 25–88 [14]. To date, there are no conclusive data concerning the exact location of the PEDV RBD and the key amino acids that participate in receptor binding.

The major genetic variations of coronaviruses have been reported to be concentrated in the S1 portion of the S gene. Even a single amino acid mutation can change the virulence of a given virus [7,19]. The sequences of the PEDV S1 domain differ between the prototype and variant strains, especially at the N-terminus (Figure 1B). For example, a six-amino-acid insertion was identified in the variant strain (residues 56–60 and 141) [20,21]. Amino acid deletions have also been identified within the S1 domain (residues 155, 156, 163, and 164) [13,22]. Whether the mutated amino acids are located within the RBD and are capable of affecting the binding affinity to its receptor and then affecting the virulence or pathogenicity is unknown.

In this study, the RBD of the PEDV S1 domain was identified, and the receptor-binding activity was compared between the variant strain CHGD-01 and the prototype CV777 strain. The motifs responsible for receptor binding were predicted and verified using mutagenesis. Finally, the sugar-binding domain of PEDV was determined, and the binding activity was compared between the CHGD-01 and CV777 strains.

## 2. Materials and Methods

### 2.1. Cell Lines and Virus Strains

Insect cells were cultured in Sf-900 II SFM (Invitrogen, Life Technologies, Carlsbad, CA, USA) at 27 °C. HEK-293T cells were grown in RPMI 1640 medium (Gibco, Waltham, MA, USA) supplemented with 10% fetal bovine serum at 37 °C with 5% CO_2_. CV777 is a PEDV prototype strain [11], and CHGD-01 is a PEDV variant field isolate that was isolated in China in 2011 [21].

### 2.2. Cloning the PEDV S1 and S1 C-Terminus Mutated Fragments

The S1 sequences of the CHGD-01 strain (residues 1–793; GenBank accession No. JN980698.1) and the CV777 strain (residues 1–789; GenBank accession No. JN599150.1) were codon-optimized and synthesized (GenScript, Nanjing, China). All of the PEDV S1-truncated variants used in this study were generated based on those templates. The S1 fragments of CHGD-01 containing residues 19–638, 1–434, 253–533, 253–638, and 477–629, which were designed based on previous studies for PEDV, TGEV, and PRCV RBDs [2,14,18], were cloned into the pFastBac1 vector with a C-terminal IgG_4_ Fc tag and an N-terminal honeybee melittin signal peptide. The S1 fragment of CV777 containing residues 249–634 was cloned in the same way. Meanwhile, the IgG_4_ Fc fragment was cloned into the pFastBac1 vector. The full-length S1 and the S1 truncated fragments of CHGD-01 containing residues 1–543, 1–434, 1–324, 1–252, 253–631, and 477–629, which were designed based on previous studies for PEDV and TGEV [2,14], were cloned into the pFastBac1 vector with a C-terminal His_6_ tag and an N-terminal honeybee melittin signal peptide. The full-length S1 and the S1 truncated fragment of CV777 containing residues 1–320 were cloned in the same way. The pAPN ectodomain (residues 63–963; GenBank accession no. HQ824547.1) was amplified and cloned into the pFastBac1 vector with a C-terminal His_6_ tag and an N-terminal honeybee melittin signal peptide.

The residues 521–534, 556–563, and 607–615 from the CHGD-01 S1 C-terminus (residues 477–629) were singly mutated to the HCoV-NL63 sequences corresponding to residues 493–506, 534–541, and 585–593, respectively; alternatively, these residues were singly mutated to the “SGSGS” motif. Residues 493–513, 531–541, and 585–590 of the HCoV-NL63 RBD form three loops that are responsible for its ACE2 binding but that do not have pAPN-binding activity [4]. “SGSGS” is a flexible linker that cannot bind pAPN. Substituting one of the three PEDV putative loops with the homologous loop of the HCoV-NL63 RBMs or with the “SGSGS” sequence would not affect the core structure of the PEDV S1 C-terminus. The primers used for the mutagenesis are provided in Supplemental Table 1. Overlap-extension PCR was performed to amplify the mutant fragments, and the PCR products were cloned into the pFastBac1 vector with a C-terminal IgG_4_ Fc tag and an N-terminal honeybee melittin signal peptide.

### 2.3. Protein Expression and Purification

The PEDV S1 fragment proteins and IgG_4_ Fc proteins were expressed and purified using a previously described protocol [3]. Briefly, various fragments of the PEDV S1 domains containing a C-terminal human IgG_4_ Fc region were expressed in insect cells using the Bac-to-Bac expression system (Invitrogen, Life Technologies, Carlsbad, CA, USA). The cell culture supernatants were harvested for protein purification using a HiTrap Protein G HP column (GE Healthcare, Uppsala, Sweden). Then, a Superdex 200 pg gel filtration column (GE Healthcare) was applied. The same procedure was used for protein expression of the C termini of various fragments of the PEDV S1 domains containing His_6_ tags, a HisTrap HP column (GE Healthcare) and a Superdex 200 pg gel filtration column were used for purification. The pAPN ectodomain containing a C-terminal His_6_ tag was expressed and purified using the same procedure as that used for the His_6_-tagged PEDV S1 domains.

### 2.4. Enzyme-Linked Immunosorbent Assay (ELISA)

The binding affinity of pAPN with different fragments of the PEDV S1 or PEDV S1 C-terminus mutated proteins was detected by ELISA [7,23]. Briefly, ELISA plates were coated with various fragments of the IgG_4_ Fc-tagged PEDV S1 or mutant proteins (60 μg/mL or different concentrations, including 120, 60, 30, 15, 7.5, 3.75, and 1.875 μg/mL) overnight at 4 °C. After washing, the plates were blocked with bovine serum albumin (BSA) for 2 h at room temperature (RT). The plates were washed and incubated with His_6_-tagged pAPN protein (50 μg/mL) for 2 h at RT. Mouse anti-His monoclonal antibody (1:5000, Proteintech, Wuhan, China) was added to the plates after washing and was incubated for 1 h at 37 °C. After washing, the plates were incubated with horseradish peroxidase (HRP)-conjugated goat anti-mouse IgG at 37 °C for 45 min (1:5000, Boster, Wuhan, China). The 3,3′,5,5′-tetramethylbenzidine (TMB) substrate was added after the incubation. The reaction continued for 7 min and was subsequently stopped by adding 50 μL of 0.25% HF. The ELISA signal was measured using the ELISA plate reader SpectraMax 190 (Molecular Devices, Sunnyvale, CA, USA).

To detect the binding affinity between sugars and different PEDV S1 domain fragments, ELISA plates were coated with 60 μg/mL bovine submaxillary gland mucin (BSM) at 4 °C overnight. After washing, the plates were blocked with BSA for 2 h at RT and incubated with 1 μM of the different fragments of the His_6_-tagged PEDV S1 domain. After washing, the ELISA was performed as described above [6].

### 2.5. Dot-Blot Hybridization Assay

Receptor-binding assays of the PEDV S1 fragment proteins were performed as previously described [7]. Briefly, for the dot-blot overlay assay, 6 μg of IgG_4_ Fc-tagged PEDV S1 fragment proteins and S1 C-terminus mutant proteins were spotted onto nitrocellulose membranes. The membranes were then completely dried, blocked with BSA at 4 °C overnight and incubated with 20 μg/mL His_6_-tagged pAPN for 4 h at RT. After washing three times with TBST, the blots were incubated with mouse anti-His monoclonal antibody at RT for 2.5 h. After another three washes with TBST, the blots were incubated with HRP-conjugated goat anti-mouse IgG antibody (1:5000, Boster) at RT for 2 h. Finally, the results were detected using a chemiluminescence reagent (ECL, Beyotime, Shanghai, China) and visualized with the G: Box Chemi XT4 (Syngene, Cambridge, UK).

### 2.6. Pull-Down Assay

The protein pull-down assay was performed as previously described [24]. Briefly, 25 μg of His_6_-tagged pAPN was mixed with 25 μg of each of the IgG_4_ Fc-tagged PEDV S1 fragment proteins and S1 C-terminus mutant proteins. The formed complexes were precipitated using Ni Sepharose™ 6 Fast Flow (GE Healthcare) by incubation for 1 h at 4 °C. After washing with RIPA lysis buffer, the His_6_-tagged pAPN and IgG_4_ Fc-tagged PEDV S1 fragment proteins were separated by SDS-PAGE and detected using anti-His antibodies and anti-human IgG_4_ Fc antibodies (1:500, Abcam, Cambridge, UK), respectively.

### 2.7. Flow Cytometry Analysis

HEK-293T cells were transfected with a pAPN gene-containing plasmid to obtain receptor-expressing cells 48 h prior to their use in a binding assay. The cells (1 × 10^6^) were then harvested with PBS containing 5 mM EDTA and were aliquoted into microcentrifuge tubes. The pellets were subsequently resuspended in PBS containing 10% fetal bovine serum (FBS) and various fragments of IgG_4_ Fc-tagged PEDV S1 proteins. The cells and proteins were incubated for 1 h at RT and washed once in PBS with 2% FBS. The pellets were then resuspended in 100 μL of PBS with 2% FBS containing FITC-labeled anti-human IgG_4_ Fc antibodies (Abcam, Cambridge, UK) and incubated for 1 h at 4°C. Following this, fluorescence-activated cell sorting (FACS) was performed using a FACScan instrument (Becton Dickinson, Franklin Lakes, NJ, USA).

### 2.8. Sequence Alignment and Structure Model Prediction

The CHGD-01 (residues 477–629), TGEV (residues 507–650; GenBank accession no. ABG89335.1) and HCoV-NL63 (residues 481–616; GenBank accession no. AFV53148.1) RBD sequences were aligned using ClustalW. The structure model of the PEDV S1 C-terminus motif was predicted using SWISS-MODEL and the PRCV S protein was chosen as a template for modeling, then compared PEDV S1 C-terminus with the RBD structures of TGEV (PDB code No. 4F2M) and HCoV-NL63 (PDB code no. 3KBH).

## 3. Results

### 3.1. The RBD Is Located in the C-Terminal Region of the PEDV S1 Domain

**Figure 2:**
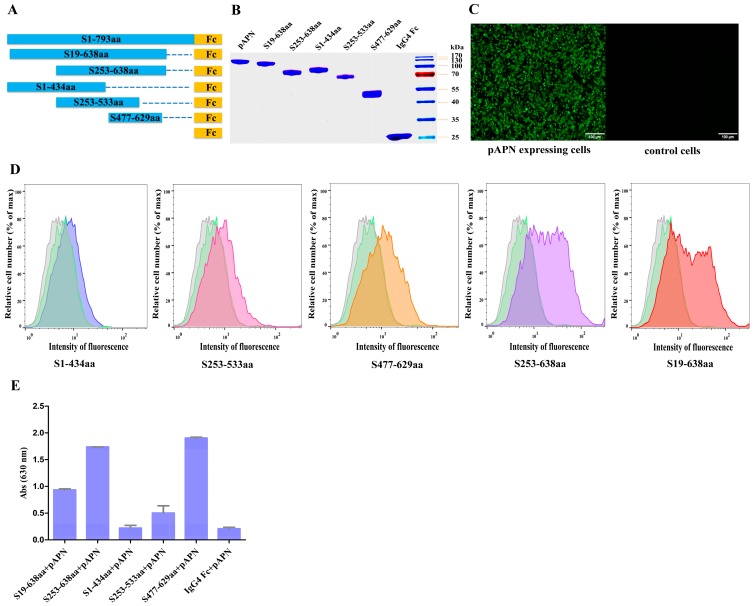
Localization of the receptor-binding region of PEDV S1. (**A**) Diagram of the codon-optimized full-length S1 and the five truncated S1 variant constructs of the CHGD-01 strain, including the S_1__9–__638_ fragment, which contains the S1 NTD and CTD; the S_1–434_ fragment, which contains the S1 NTD; the S_253–533_ fragment; the S_477–629_ fragment; and the S_253__–__638_ fragment, which is a CTD of S1; (**B**) Purified truncated S1 variants of CHGD-01 with a C-terminal IgG_4_ Fc tag, pAPN with a C-terminal His_6_ tag, and IgG_4_ Fc proteins were detected by SDS-PAGE; (**C**) The transfection efficiency was determined by performing an indirect immunofluorescence assay. HEK-293T cells transfected with a pAPN gene-containing plasmid (**left**) and cells transfected with an empty vector (**right**) were used for detection. Mouse anti-His monoclonal antibody and Alexa Fluor^®^ 488 anti-mouse IgG (H + L) antibody were used before detection. The green fluorescence represents the expressed pAPN protein; (**D**) The binding between five truncated S1 variants and pAPN-expressing cells was investigated by performing flow cytometry. HEK-293T cells were transfected with a pAPN gene-containing plasmid. After 48 h, the cells were resuspended with the S_19__–__638_, S_253__–__638_, S_1__–__434_, S_253__–__533_, and S_477__–__629_ proteins (50 μg/mL), followed by treatment with FITC-labeled anti-human IgG_4_ Fc antibodies. Then FACS was performed. pAPN-transfected cells untreated with protein (grey shade) or treated with IgG_4_ Fc protein (green shade) were used as controls; (**E**) An ELISA was performed to detect the binding between five truncated S1 variants and pAPN, IgG_4_ Fc protein was used as a control. ELISA plates were coated with S_19__–__638_, S_253__–__638_, S_1__–__434_, S_253__–__533_, S_477__–__629_, or IgG_4_ Fc proteins and blocked with BSA. The plates were then incubated with the pAPN protein. The TMB substrate was added after incubation with mouse anti-His monoclonal antibody and HRP-conjugated goat anti-mouse IgG antibody. The experiment was carried out three times, and the data from a representative experiment are shown. Error bars indicate the standard errors of the means.

To identify the RBD in the PEDV S1 subunit, we constructed five truncated expression plasmids (S_19–638_, S_253–638_, S_1–434_, S_253–533_, S_477–629_) of the CHGD-01 strain with a C-terminal IgG_4_ Fc tag (Figure 2A). A plasmid expressing the IgG_4_ Fc protein was constructed as a control. A plasmid that encoded pAPN with a C-terminal His_6_ tag was also constructed. These proteins were expressed in insect cells and purified using Protein G or HisTrap HP columns. As shown in Figure 2B, highly purified proteins were obtained.

Flow cytometry was conducted to evaluate pAPN-expressing cells’ binding of the S_19–638,_ S_253__–__638_, S_1–434_, S_253–533_, and S_477–629_ fragments. pAPN-transfected cells untreated with protein or treated with IgG_4_ Fc protein were used as controls. To detect the transfection efficiency, HEK-293T cells transfected with a pAPN gene-containing plasmid were used in an indirect immunofluorescence assay, our result showed that the pAPN protein was highly expressed on cell surface (Figure 2C). The flow cytometry assay results showed that the S_19__–__638_ and S_253__–__638_ fragments revealed the highest binding affinity, the S_477–629_ fragment exhibited moderate pAPN-binding activity, and the S_1–434_ and S_253–533_ fragments revealed weak binding affinity, whereas the IgG_4_ Fc protein revealed no binding affinity (Figure 2D). These results suggest that the pAPN-binding domain is located within the CTD of the PEDV S1 domain.

An ELISA was also performed to examine the associations between the S1 fragments and pAPN. The results showed that the S_19__–__638_, S_253__–__638_, and S_477–629_ fragments bound to pAPN with high affinity, while the S_253–533_ fragment bound to pAPN with very low affinity. In contrast, the S_1–434_ fragment and IgG_4_ Fc protein did not bind to pAPN (Figure 2E). These results further confirmed that the RBD is located within the CTD of the PEDV S1 domain.

Taken together, we mapped the RBD of the PEDV S protein to the CTD of the S1 domain by performing flow cytometry and ELISA assays.

### 3.2. The Prototype Strain Shows Similar Receptor-Binding Activity as the Variant Field Isolate

**Figure 3:**
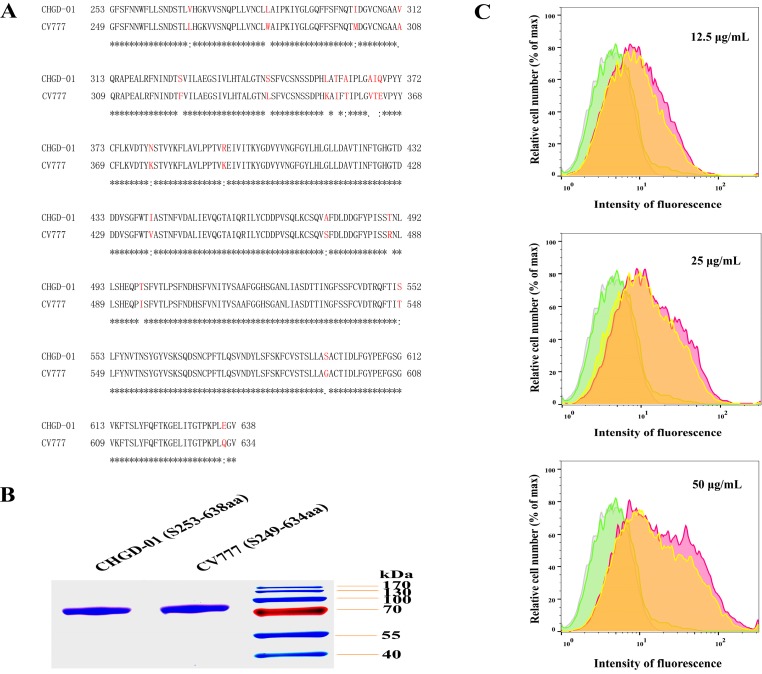
Comparison of pAPN-binding activity between CV777 and CHGD-01. (**A**) Sequence alignment of the CHGD-01 S_253–__638_ and the CV777 S_249–__634_ was carried out by ClustalW. The different residues are shown in red; (**B**) Purified proteins of the CV777 S_249–__634_ and the CHGD-01 S_253–__638_ with a C-terminal IgG_4_ Fc tag were detected by SDS-PAGE; (**C**) The binding of the CV777 S_249–__634_ or the CHGD-01 S_253–__638_ with pAPN-expressing cells was investigated by flow cytometry. HEK-293T cells were transfected with a pAPN gene-containing plasmid. After 48 h, the cells were resuspended with different concentrations of the CHGD-01 S_253__–__638_ protein (pink shade) or the CV777 S_249__–__634_ protein (yellow shade), including 12.5 μg/mL (**top**), 25 μg/mL (**middle**), and 50 μg/mL (**bottom**), followed by treatment with FITC-labeled anti-human IgG_4_ Fc antibodies. Then FACS was performed. pAPN-transfected cells untreated with protein (grey shade) or treated with IgG_4_ Fc protein (green shade) were used as controls.

Sequence comparison of the CHGD-01 S_253__–__638_ fragment and the CV777 S_249–__634_ fragment found that they exhibited very high similarity, with 94.6% homology (Figure 3A). To compare the receptor-binding activity between the variant field isolate and the prototype strain, a plasmid expressing residues 249–634 of the S1 domain from the CV777 strain was constructed with a C-terminal IgG_4_ Fc tag. The protein was expressed in insect cells and purified with a Protein G column; a highly purified protein was obtained (Figure 3B). Then, the receptor-binding activities of the CHGD-01 S_253__–__638_ fragment and the CV777 S_249–__634_ fragment were investigated using a flow cytometry assay, different concentrations of the CHGD-01 S_253__–__638_ or the CV777 S_249–__634_ protein including 12.5, 25, and 50 μg/mL were used for detection. pAPN-transfected cells untreated with protein or treated with IgG_4_ Fc protein were used as controls. The result showed that the S_253–__638_ fragment of CHGD-01 exhibited similar pAPN-binding activity with the S_249–__634_ fragment of CV777 (Figure 3C).

### 3.3. Structural and Sequence Similarities of the PEDV S1 C-Terminus with TGEV, PRCV, and HCoV-NL63 RBDs

**Figure 4:**
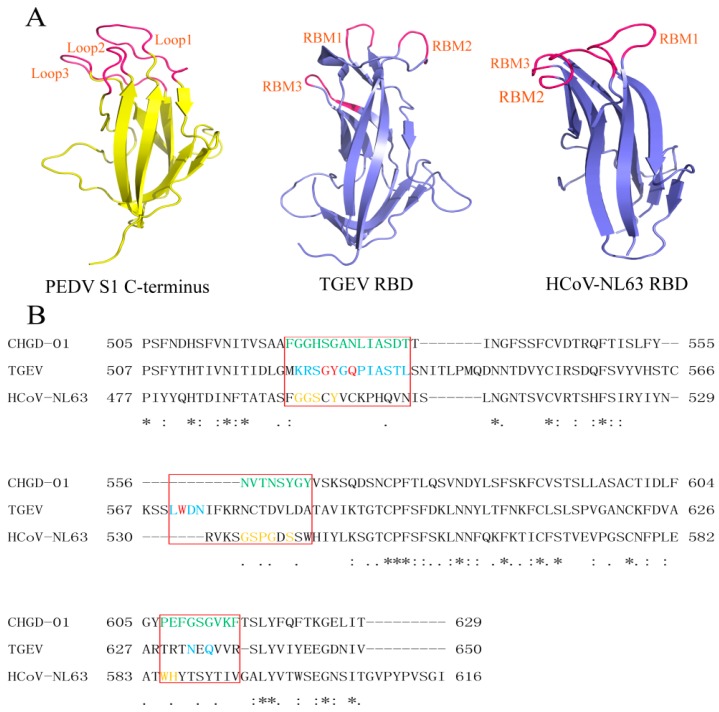
Structural and sequence comparison between the PEDV S1 C-terminus and the TGEV and HCoV-NL63 RBDs. (**A**) Structural comparison of the PEDV S1 C-terminus (**left**), TGEV RBD (**middle**), and HCoV-NL63 RBD (**right**). The PEDV S1 C-terminus is shown in yellow, the TGEV and HCoV-NL63 RBDs are shown in slate, and the motifs responsible for receptor binding are marked in hot pink; (**B**) Structure-based sequence alignments of the PEDV S1 C-terminus, TGEV RBD, and HCoV-NL63 RBD. Residues boxed in red are RBMs for TGEV and HCoV-NL63 or are predicted RBMs for PEDV; these motifs correspond to the three loops at the tips of the β-barrel domains of the three coronaviruses. PEDV mutated residues are colored in green. TGEV RBD and pAPN residues in close contact are marked in cyan, and those engaged in hydrogen bonding are shown in red. Residues on HCoV-NL63 RBD that are in direct contact with ACE2 are marked in orange.

To investigate the similarities and differences in receptor recognition between PEDV and other coronaviruses, we predicted the structure of the PEDV S1 C-terminus (residues 477–629), which corresponds to the sequences of TGEV, PRCV, and HCoV-NL63 RBDs using SWISS-MODEL. The PEDV S1 C-terminus was predicted to contain eight β-strands and several loops (Figure 4A, left). Importantly, three loops were located at the tips of the β-barrel domains in the predicted structure; these loops also occur in the structure of TGEV RBD (Figure 4A, middle) and the HCoV-NL63 RBD (Figure 4A, right). Then, the correspondence sequences of PEDV, TGEV, and HCoV-NL63 were aligned using ClustalW. The results showed that the three loops responsible for receptor binding at the tips of the β-barrel domains of PEDV, TGEV, and HCoV-NL63 were located at the same site (Figure 4B). Since PEDV, TGEV, PRCV, and HCoV-NL63 all belong to *Alphacoronavirus*, our results confirmed that the RBD of PEDV was located in the CTD. Based on the above results, we infer that the three loops at the tips of the β-barrel domains may also be responsible for PEDV receptor binding.

### 3.4. PEDV Conforms to a Different Receptor Recognition Model Compared with TGEV, PRCV, and HCoV-NL63

**Figure 5:**
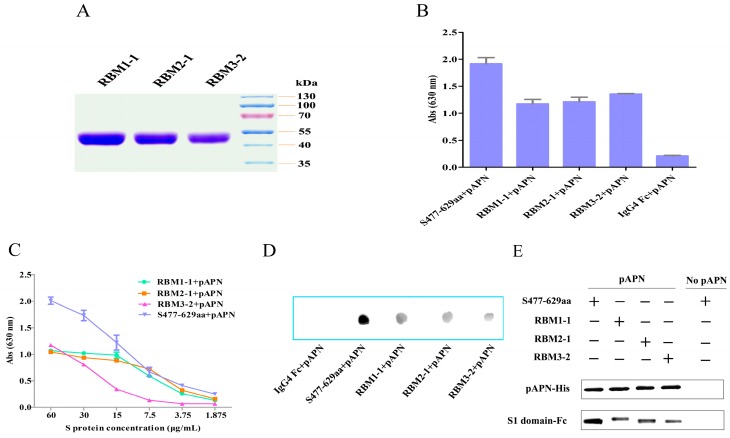
The effect of mutation of the three loops on pAPN binding. (**A**) Purified RBM1-1, RBM2-1, and RBM3-2 proteins with a C-terminal IgG_4_ Fc tag were detected by SDS-PAGE; (**B**) An ELISA was performed to detect the binding between the CHGD-01 S_477–629_, RBM1-1, RBM2-1, RBM3-2 (60 μg/mL), and pAPN. The IgG_4_ Fc protein was used as a control. ELISA plates were coated with the CHGD-01 S_477__–629_, RBM1-1, RBM2-1, RBM3-2, and IgG_4_ Fc proteins and blocked with BSA. The plates were then incubated with the pAPN protein. The TMB substrate was added after incubation with mouse anti-His monoclonal antibody and HRP-conjugated goat anti-mouse IgG antibody. The experiment was carried out three times, and the data from a representative experiment are shown. Error bars indicate the standard errors of the means; (**C**) Different concentrations of the CHGD-01 S_477__–629_, RBM1-1, RBM2-1, and RBM3-2 proteins were used to detect the pAPN-binding activity by ELISA. The experiment was carried out three times, and the data from a representative experiment are shown; (**D**) A dot-blot assay was performed to compare the pAPN-binding activity between CHGD-01 S_477__–629_, RBM1-1, RBM2-1, and RBM3-2. The IgG_4_ Fc protein was used as a control. The CHGD-01 S_477__–629_, RBM1-1, RBM2-1, RBM3-2, and IgG_4_ Fc proteins were spotted onto nitrocellulose membranes, then blocked with BSA and incubated with pAPN. Before visualization, the blots were incubated with mouse anti-His monoclonal antibody and HRP-conjugated goat anti-mouse IgG antibody; (**E**) A pull-down assay was performed to assess the pAPN-binding activities of the CHGD-01 S_477__–629_, RBM1-1, RBM2-1, and RBM3-2. pAPN was mixed with the CHGD-01 S_477__–629_, RBM1-1, RBM2-1, and RBM3-2 proteins. The formed complexes were precipitated using Ni Sepharose™ 6 Fast Flow. After washing with RIPA lysis buffer, the proteins were separated by SDS-PAGE and detected using anti-His antibodies and anti-human IgG_4_ Fc antibodies, respectively. The top panel shows the input pAPN protein and the bottom panel shows the precipitated CHGD-01 S_477__–629_, RBM1-1, RBM2-1, and RBM3-2 proteins.

Sequence alignment showed that the PEDV S1 C-terminus (residues 477–629) corresponded to the RBDs of TGEV, PRCV, and HCoV-NL63. Coincidentally, the S_477–629_ fragment from CHGD-01 strain exhibited pAPN-binding activity in the ELISA and flow cytometry assays. To determine whether PEDV uses the same receptor recognition model as TGEV, PRCV, and HCoV-NL63, the three loops from the CHGD-01 S_477–629_ fragment were individually replaced with those of HCoV-NL63 or the sequence “SGSGS.” The mutants were then expressed in insect cells. Three proteins were successfully expressed: RBM1-1 (residues 521–534 were mutated to the HCoV-NL63 sequence corresponding to residues 493–506), RBM2-1 (residues 556–563 were mutated to the HCoV-NL63 sequence corresponding to residues 534–541), and RBM3-2 (residues 607–615 were mutated to “SGSGS”). These proteins were purified by Protein G affinity chromatography (Figure 5A). An ELISA was performed to detect the binding activities between these mutant proteins and the pAPN protein. The receptor-binding activities of the three mutant proteins showed 30%–40% reduction compared with that of the wild-type S_477–629_ fragment protein (Figure 5B). The same results were obtained when different concentrations (including 60, 30, 15, 7.5, 3.75, and 1.875 μg/mL) of the RBM1-1, RBM2-1, and RBM3-2 proteins were used for the ELISA (Figure 5C).

The dot-blot hybridization assay was used to compare the pAPN-binding activities between the wild-type and the three mutant proteins. The results revealed that the RBM1-1, RBM2-1, and RBM3-2 proteins did not completely lose their pAPN-binding activities (Figure 5D).

We also performed a pull-down assay to compare the pAPN-binding activities of the wild-type and the three mutant proteins. The results demonstrated that the RBM1-1, RBM2-1, and RBM3-2 proteins retained their pAPN-binding activities, although these pAPN-binding activities were not as strong as for the wild-type S_477–629_ fragment protein (Figure 5E). Collectively, the ELISA, dot-blot hybridization, and pull-down assay results suggest that the three loops at the tips of the β-barrel domains did not play a crucial role in the PEDV S-pAPN association. Thus, PEDV S-pAPN recognition conforms to a different model compared with TGEV, PRCV, and HCoV-NL63. 

### 3.5. The Sugar-Binding Site Is Located in the N-Terminal Region of the S1 Domain, and the Sugar-Binding Activity of the Prototype Strain Is Weaker than That of the Variant Field Isolate

**Figure 6:**
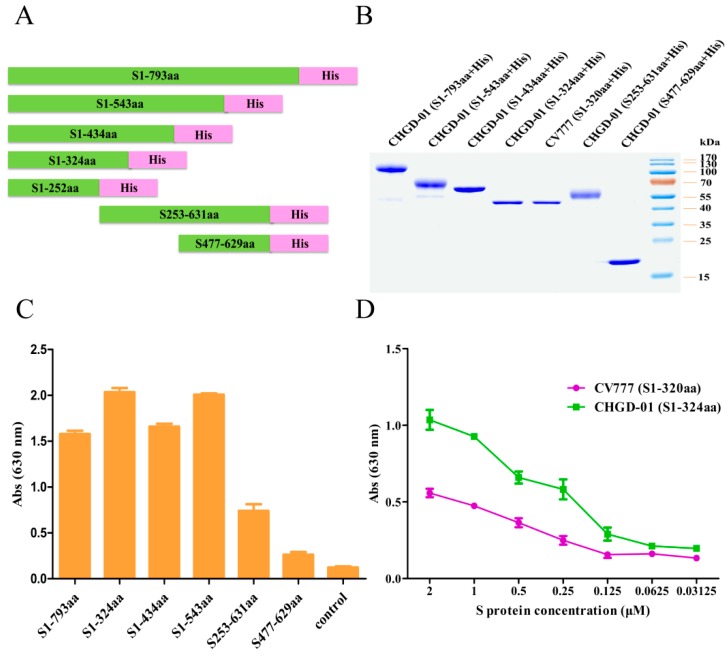
Localization of the sugar-binding region of PEDV S1. (**A**) Diagram of the codon-optimized full-length S1 and six truncated S1 variants of the CHGD-01 strain; (**B**) Purified full-length S1 and truncated S1 variants of the CHGD-01 and CV777 strains with a C-terminal His_6_ tag were detected by SDS-PAGE; (**C**) An ELISA was performed to detect the binding between the full-length S1, five truncated S1 variants of CHGD-01 strain, and mucin. Wells without S fragment protein were used as controls. ELISA plates were coated with the S_1–793_, S_1__–543_, S_1__–434_, S_1__–324_, S_253__–631_, and S_477__–629_ proteins and blocked with BSA. The plates were then incubated with the pAPN protein. The TMB substrate was added after incubation with mouse anti-His monoclonal antibody and HRP-conjugated goat anti-mouse IgG antibody. The experiment was carried out three times, and the data from a representative experiment are shown. Error bars indicate the standard errors of the means; (**D**) The mucin-binding activities of the S_1__–324_ fragment from the CHGD-01 strain and the S_1__–320_ fragment from the CV777 strain were compared by ELISA. The experiment was carried out three times, and the data from a representative experiment are shown.

To determine whether the PEDV S protein possesses sugar-binding activity, we constructed a full-length S1 plasmid and six truncated S1 plasmids for the CHGD-01 strain. The plasmids encoded S_1–793_, S_1__–543_, S_1__–434_, S_1__–324_, S_1__–252_, S_253__–631_, and S_477__–629_ with a C-terminal His_6_ tag (Figure 6A), and these proteins were expressed in insect cells. All of the proteins were successfully expressed, with the exception of the S_1__–252_ truncation, which showed a very low level of expression. The highly expressed proteins were purified using a HisTrap HP column (Figure 6B). Then, ELISA was performed to detect their binding interactions with bovine mucin, a mixture of highly glycosylated proteins recognized by coronavirus S proteins and containing all of the sugar moieties including 5-*N*-acetyl-9-*O*-acetylneuraminic acid (Neu5,9Ac2), 5-*N*-glycolylneuraminic acid (Neu5Gc), and 5-*N*-acetylneuraminic acid (Neu5Ac). The results showed that the S_1–793_, S_1__–324_, S_1__–434_, and S_1__–543_ fragments bound to mucin with high affinity, while the S_253__–631_ fragment bound to mucin with a much lower affinity, and the S_477__–629_ fragment had little binding activity (Figure 6C). The results demonstrate that the N-terminal region of the PEDV S1 domain possesses sugar-binding activity.

To compare the sugar-binding activities between the PEDV variant field isolate and the prototype strain, full-length S1 and the S_1__–320_ truncation of the CV777 strain were constructed with C-terminal His_6_ tags. These proteins were expressed and purified using a HisTrap HP column. The S_1__–320_ truncation was successfully expressed and purified, whereas, expression of the full-length S1 domain gave low yields (Figure 6B). Then, an ELISA was performed to detect the mucin-binding activities of the CV777 S_1__–320_ fragment and the CHGD-01 S_1__–324_ fragment. The results showed that the S_1__–324_ fragment from the CHGD-01 strain had stronger binding activity than that of the S_1__–320_ fragment from the CV777 strain (Figure 6D), indicating that the sugar-binding activity of the PEDV prototype strain is weaker than that of the variant field isolate.

## 4. Discussion

Coronavirus cell entry and interspecies transmission are mediated by the S protein [25]. The coronavirus S protein can recognize several proteins and sugars as receptors or co-receptors [6]. The receptor recognition mechanisms are diverse among different coronaviruses. The crystal structure of the SARS-CoV RBD and ACE2 complex revealed a continuous receptor-binding motif located at residues 424–494 of the S1 domain [26]. In contrast, the CTD of HCoV-NL63 S1 is composed of a β-sandwich structure that contains three discontinuous receptor-binding motifs responsible for ACE2 binding [4]. The crystal structures of the PRCV and TGEV RBDs show a single domain unit that adopts a β-barrel fold with two highly twisted β-sheets located in the CTD of the S1 domain and participates in pAPN binding [2]. The NTD of the MHV S1 domain is a RBD for the CEACAM interaction, and the crystal structure of the MHV NTD shows a galectin-like fold with four discontinuous receptor-binding motifs [7]. The homologous BCoV NTD also possesses a galectin fold but uses Neu5,9Ac2 as its receptor [6]. Several studies have identified pAPN as a functional receptor for PEDV virus entry [16,17], but the exact location of the RBD and the function of the NTD and CTD of the S1 domain in receptor recognition have not yet been determined.

Previous studies have identified PC177 strain with a 197-amino-acid deletion and Tottori2 strain with a 194-amino-acid deletion in the N-terminus of the S protein that could propagate in Vero cells [27,28], indicating that the RBD of PEDV may not be located in the N-terminus of the S1 domain. RBD sequences and structures vary considerably among different coronaviruses, and they use distinct receptors for virus entry [29]. The RBDs of most coronaviruses are located in the CTD of the S1 domain, especially for *Alphacoronaviruses,* including TGEV, PRCV, HCoV-229E, and HCoV-NL63. The RBDs from these viruses are all located near the C-terminal region of the S1 domain [29,30]. Our results are in agreement with previous studies. Hence, we can conclude that the pAPN-binding domain of the PEDV S1 domain is located within its CTD. In Figure 2D,E, the results of the S_19–638_ and S_477–629_ fragments were not consistent. The reason for this discrepancy is not clear but may be related to the different exposing level of protein surface in ELISA and FACS.

In the crystal structure of the PRCV RBD-pAPN complex, three loops (β1–β2, β3–β4, and β5–β6) at the tips of the β-barrel domains are responsible for receptor binding [2]. Single amino acid mutations in the three loops completely or significantly reduce PRCV RBD binding to pAPN, and the mutations outside the RBD have no effect on receptor recognition [2]. The crystal structure of the HCoV-NL63 RBD-ACE2 complex shows three protruding β-loops surrounding a shallow bowl-shaped cavity at the top of the RBD that are important for receptor binding. RBM3 (residues 585–590) is more compact than either RBM1 (residues 493–513) or RBM2 (residues 531–541) [4]. Only one amino acid mutation completely or greatly reduced the ACE2 binding activity of HCoV-NL63 [31]. In our study, the RBM1-1, RBM2-1, and RBM3-2 proteins did not significantly lose their pAPN-binding activities, indicating that the PEDV S protein uses a different receptor-binding model than TGEV, PRCV, and HCoV-NL63, which was further confirmed by PEDV displaying a broader receptor binding region than other *Alphacoronaviruses* in our study.

Coronaviruses use a variety of cellular molecules as receptors or co-receptors, including proteins and sugars. A sugar moiety on cell-surface glycoproteins or glycolipids (*i.e.*, Neu5,9Ac2) can be recognized by BCoV and HCoV-OC43 in the genus *Betacoronavirus* [6,32]. Moreover, two other types of sugars (Neu5Gc and Neu5Ac) can serve as receptors or co-receptors for some *α*- and *Gammacoronaviruses*, such as TGEV and IBV [9,33,34]. The NTD of the TGEV S1 domain is responsible for its enteric tropism, and the related PRCV, which lacks this domain, does not show enteric tropism [35]. Thus, binding sugar as a co-receptor is linked with the enteric tropism of coronaviruses [36]. In the present study, a flow cytometry assay found that the S_1__–__434_ and S_253__–__533_ fragments could weakly bind to pAPN-expressing cells, and that the S_19–638_ fragment revealed a much higher binding affinity, which indicates that except for pAPN recognized by the CTD of the PEDV S1 domain, other cellular molecules, such as sugars, can be recognized by the NTD of the S1 domain as receptors or co-receptors. Similar to TGEV, which uses Neu5Gc and Neu5Ac as co-receptors [33,36,37], we confirmed that the NTD of the PEDV S1 domain can also interact with sugar. Thus, sugar may act as a co-receptor for PEDV.

The variations in coronaviruses are concentrated in the S1 domain [29]. Sequence analysis of PEDV prototype and variant strains revealed more than 100 amino acid mutations within the S protein, especially in the N-terminus, but very few changes are found within the C-terminus [20,21]. When comparing the RBD sequences between the CHGD-01 and the CV777 strains, only several amino acid mutations were found, so it is reasonable that the pAPN-binding activities of the CHGD-01 and CV777 RBDs are similar. When the sugar-binding activity was compared between the CHGD-01 and the CV777 strains, the CHGD-01 strain showed stronger binding efficiency. This finding is not unreasonable due to the obvious difference in the N-terminal S1 gene sequences between the two strains. Most likely, the mutated amino acids participate in sugar binding. Furthermore, among the many factors that contribute to the infectivity and pathogenicity of PEDV, the enhanced receptor binding activity of the variant strain may play important roles. Prior to 2010, PEDV infection was relatively mild, with sporadic PED outbreaks; however, since late 2010, PED outbreaks have occurred in many countries in Asia and North America [13,38]. Further studies are warranted to investigate whether the enhanced receptor binding activity of the new variant strain is responsible for the recent PED outbreaks.

## 5. Conclusions

In summary, we demonstrated that the C-terminal region of the PEDV S1 domain is responsible for binding to pAPN as its major receptor and it conforms to a different receptor recognition model compared with TGEV, PRCV, and HCoV-NL63. The N-terminal region of the PEDV S1 domain is responsible for binding to sugar, which may act as its co-receptor. The sugar-binding activities of the variant field isolate are stronger than those of the prototype strain. The first and crucial step of virus infection is virus–receptor interaction; thus, investigations on receptor recognition are important for understanding the mechanisms of virus invasion and pathogenesis and will provide rational strategies for developing antiviral drugs, vaccines, and monoclonal antibodies for the treatment and prevention of viral infections.

## Supplementary Files

Supplementary File 1
