# Supplementary material for: Identification and Comparison of Receptor Binding Characteristics of the Spike Protein of Two Porcine Epidemic Diarrhea Virus Strains
Source: Viruses. 2016 Feb 23;8(3):55. doi: 10.3390/v8030055 (PMC4810246; doi:10.3390/v8030055)
Supplement: Supplementary File 1 [file viruses-08-00055-s001.pdf]

Supplementary Materials: Identification and Comparison of Receptor Binding Characteristics of the Spike Protein of Two Porcine Epidemic Diarrhea Virus Strains

Feng Deng, Gang Ye, Qianqian Liu, Muhammad Tariq Navid, Xiaoli Zhong, Youwen Li, Chunyun Wan, Shaobo Xiao, Qigai He, Zhen F. Fu and Guiqing Peng

Table S1. Primers used for RBD mutations.

Primers	Primer Sequence (5′–3′)	Mutations
CHGD-01-RBM1-R1	TGGTTTACAAACATAACAAGAACCACCAAAGGC AGCTGAGACGGTGAT	RBM1-1 [a]
CHGD-01-RBM1-F1	TGTTATGTTTGTAAACCACACCAGGTTAATACCA TCAACGGTTTCTCT	RBM1-1 [a]
CHGD-01-RBM2-R1	CCATGAAGAGTCACCTGGTGAACCGTAGAACAA GCTGATTGT	RBM2-1 [a]
CHGD-01-RBM2-F1	GGTTCACCAGGTGACTCTTCATGGGTGTCCAAGT CTCAGGAC	RBM2-1 [a]
CHGD-01-F	CCGGAATTCATGAAATTCTTAGTCAACGTTGCCC TTGTTTTTATGGTCGTGTACATTTCTTACATCTATG CGGCCTTCGACCTCGATGAC	RBM3-1 [a]
CHGD-01-RBM3-R1	AGTGATCAGCTCTCCCTTTGTGAACTGGAAGTAC AGCGAAGTAACAATAGTATAAGAAGTGTAATGC CAGTAGCCGAACAAGTCGAT	RBM3-1 [a]
CHGD-01-F [c]	CCGGAATTCATGAAATTCTTAGTCAACGTTGCCC TTGTTTTTATGGTCGTGTACATTTCTTACATCTATG CGGCCTTCGACCTCGATGAC	
CHGD-01-R [d]	CGGGGTACCGCTGCCGCTAGTGATCAGCTCTC	
CHGD-01-RBM1-R2	GCTGCCGCTGCCGCTGGCAGCTGAGACGGTGAT	RBM1-2 [b]
CHGD-01-RBM1-F2	AGCGGCAGCGGCAGCACCATCAACGGTTTCTCT	RBM1-2 [b]
CHGD-01-RBM2-R2	GCTGCCGCTGCCGCTGTAGAACAAGCTGATTGT	RBM2-2 [b]
CHGD-01-RBM2-F2	AGCGGCAGCGGCAGCGTGTCCAAGTCTCAGGAC	RBM2-2 [b]
CHGD-01-F	CCGGAATTCATGAAATTCTTAGTCAACGTTGCCC TTGTTTTTATGGTCGTGTACATTTCTTACATCTATG CGGCCTTCGACCTCGATGAC	RBM3-2 [b]
CHGD-01-RBM3-R2	AGTGATCAGCTCTCCCTTTGTGAACTGGAAGTAC AGCGAAGTGCTGCCGCTGCCGCTGTAGCCGAAC AAGTCGAT	RBM3-2 [b]

F, F1, F2, forward; R, R1, R2, reverse; [a] RBM1, RBM2 and RBM3 of the CHGD-01 S1 were mutated to the corresponded sequences of the HCoV-NL63 S1; [b] RBM1, RBM2 and RBM3 of the CHGD-01 S1 were mutated to the "SGSGS" motif; [c], [d] primers used for overlap extension PCR for the RBM1 and RBM2 mutations.

© 2016 by the authors; licensee MDPI, Basel, Switzerland. This article is an open access article distributed under the terms and conditions of the Creative Commons by Attribution (CC-BY) license (http://creativecommons.org/licenses/by/4.0/).
